# Use of Short Stems in Revision Total Hip Arthroplasty: A Retrospective Observational Study of 31 Patients

**DOI:** 10.3390/medicina59101822

**Published:** 2023-10-13

**Authors:** Marlene Mauch, Hendrik Brecht, Martin Clauss, Karl Stoffel

**Affiliations:** 1 Department of Orthopedics and Traumatology, University Hospital Basel, 4031 Basel, Switzerland; hendrik.brecht@usb.ch (H.B.); martin.clauss@usb.ch (M.C.); karl.stoffel@usb.ch (K.S.); 2 Department of Biomedical Engineering, University of Basel, 4123 Allschwil, Switzerland; 3 Center for Musculoskeletal Infections (ZMSI), University Hospital Basel, 4031 Basel, Switzerland

**Keywords:** hip replacement, surgical revision, postoperative complications, patient outcomes’ assessment

## Abstract

*Background and Objectives*: Implantation of a short femoral stem in revision total hip arthroplasty (rTHA) could reduce the perioperative time, soft tissue damage, and preserve the bone stock of the proximal femur. The objective of this study was to describe the clinical and radiographic outcomes after the use of short stems in rTHA with a follow-up of 1 to 5 years. *Materials and Methods*: This retrospective, single center, and observational study analyzed the data of 31 patients (12 female, 19 male) with a median (interquartile range) age of 68.2 years (61.2–78.4) and BMI of 26.7 kg/m^2^ (24.6–29.4) who received an uncemented short femoral stem in rTHA between 2015 and 2020. Clinical outcomes were extracted from medical reports and assessed using the modified Harris Hip Score (mHHS), the numerical rating scale (NRS) for pain and satisfaction, and the UCLA Physical Activity Score. Radiographs were analyzed for stem subsidence, fixation, and bone parameters. The Wilcoxon test was used for pre–post rTHA differences (*p* < 0.05); clinical relevance was interpreted based on effect sizes according to Cohen’s *d*. *Results*: All the clinical outcome measures improved significantly (*p* ≤ 0.001) at follow-up compared to preoperative status, with large effect sizes (Cohen’s *d*) ranging from 2.8 to 1.7. At the last follow-up, the median (interquartile) mHHS was 80.9 (58.6–93.5). Stem fixation was stable in all cases. Complications included stem subsidence of 3 mm (n = 1) and 10 mm (n = 1), heterotopic ossification Brooker stage III (n = 2), intraoperative femur perforation (n = 1), periprosthetic fracture Vancouver type A (n = 1), and dislocation (n = 2). *Conclusions*: The good clinical results in our selective study population of patients with mild to moderate bone deficiency, supported by large effect sizes, together with a complication rate within the normal range, support the consideration of short stems as a surgical option after a thorough preoperative analysis.

## 1. Introduction

Short femoral stems are becoming increasing popular in primary total hip arthroplasty (THA) using minimally invasive surgical techniques. A short stem is defined as a femoral stem component, preferably with metaphyseal fixation, with a length (center of head to prosthesis tip (CT)) of 120 mm or less [1]. While the diaphyseal connection of standard implants could potentially cause proximal stress shielding, short stems were designed to achieve a more anatomical pattern of stress distribution through the metaphyseal fixation, allowing for proximal load transfer and reducing bone resorption [2,3]. Even standard uncemented femoral stems are clinically, functionally, and radiographically effective in hip replacement surgery [4]; the use of short stems is becoming increasingly popular, allowing for bone-conserving implantation, improved proximal bone remodeling, less traumatic surgical techniques, and simplified revisions [4,5,6]. Conversely, an increased incidence of aseptic loosening, implant migration, and periprosthetic fractures has been demonstrated in association with the use of short-stem femoral components [7,8,9]. However, registry data have also shown no significant difference in mid-term survival between short and conventional uncemented femoral stems [1]. Given the expanded indications, increased life expectancy, and the overwhelming success of THA, the incidence of THA is increasing noticeably, even in younger patients. It is therefore expected that the numbers of and need for revision surgery will increase [10,11]. Due to infection, periprosthetic fracture, and instability, aseptic loosening is the leading cause of late THA failure [12].

In revision total hip arthroplasty (rTHA), reconstruction of the proximal femur and implantation of the femoral component is one of the most important steps. The literature describes the frequent use of various techniques, such as cementless and cemented implants and conventional or long stems [13,14,15]. However, in any subsequent revision, including implant removal, especially in the case of cemented stems, stem reimplantation is usually associated with a higher perioperative risk and further loss of bone stock [16]. Commonly, more distally fixed, tapered, and cementless revision stems are a successful model to achieve stable fixation by creating a conical anchorage bed [17], which is especially needed in patients with severe bone deficiency of the proximal femur. However, there are some disadvantages to this solution. The effect on the patient’s prognosis of perioperative fracture risk due to the mismatch of the physiological curvature of the femur, the proximal stress shield could be a large problem after the implantation of a standard or extended stem, causing bone resorption, osteolysis, and bone remodeling and even causing aseptic loosening after rTHA. Bone preservation is critical for hip revision, especially in younger patients at risk for further revision later in life. Implantation of a short femoral stem in revision cases with the known benefits of primary THA could reduce the perioperative time, soft tissue damage, and stress shielding of the proximal femur also in revision cases [18]. However, this indication is limited due to the mild to moderate proximal bone loss, which presents challenges and limitations in achieving sufficient metaphyseal fixation and early stability in revision of the femoral component. 

Systematic reviews have examined the outcomes of conventional cementless stems in rTHA [15]. To date, there is a paucity of published data on the clinical outcomes of using short stems as a revision component. There are some case reports on the repair of fractured stems [19,20,21], describing the benefits of reducing blood loss and the surgical time and facilitating easier femoral revision by avoiding distal cement removal in the femoral canal [22]. Therefore, the aim of this study was to describe the clinical and radiographic outcomes of the use of short stems in rTHA at 1 to 5 years of follow-up. We hypothesized that the use of short stems is a durable option in selected patients with sufficient bone quality and would result in good functional and clinical outcomes. Our results may provide surgeons with additional treatment options for femoral stem revision by using short stems in certain situations and may help improve patient care.

## 2. Materials and Methods

### 2.1. Study Design and Participants

In this retrospective observational study, patients who underwent rTHA at the University Hospital of Basel between 2015 and 2020 were descriptively analyzed. Overall, the data of 31 patients who received an uncemented short femoral stem (Optimys^®^, Mathys, Bettlach, Switzerland) for rTHA (ICD classification T84) were extracted. Their medical reports and radiographic images were reviewed at the time of the study (January to April 2023). Revision was defined as removal of the original hip prosthesis and implantation of a new hip prosthesis. Patients whose acetabular components were retained were also included. Follow-up was defined as at least 1 year after revision surgery. Patients with femoral components other than Optimys^®^ in rTHA, second revision, or documented dissent to the use of clinical data for research purposes were excluded from the analyses. The study was approved by the regional ethics committee (EKNZ-2022-02250).

### 2.2. Surgical Technique

The surgical approach for rTHA was dependent on the primary approach, the surgeon’s preference, and the indication including the extent of the revision. An anterior approach was performed in n = 6 patients, a lateral approach in n = 15 patients, and a posterior approach in n = 10 patients. Preoperative planning was based on imaging and included scoring of bone loss according to the Paprosky classification [23]. Diagnosis and preoperative planning for all patients were performed by two different experienced senior surgeons.

Patients were placed in the supine or lateral decubitus position, depending on the approach. Patients received a single preoperative antibiotic dose of a third-generation cephalosporin (n = 31) and tranexamic acid (n = 28). After exposure of the proximal femur and opening of the joint capsule, the primary prosthesis was dislocated in all cases. The stem was then knocked out with the appropriate revision instruments. If necessary, the stem was surrounded with chisels. The medullary canal was then cleaned of any remaining cement or debris. The acetabular cup was then fitted and adjusted according to the indication and findings. Acetabular revision was performed simultaneously in n = 23 hips (74%). After femoral repositioning, it was prepared with appropriate rasps for the short stem (Optimys^®^, Mathys, Switzerland). The depth and size of the stem was selected according to the preoperative planning and intraoperative landmarks. After trial fitting, the situs was rinsed, and the definitive stem was hammered in. After head replacement, clinical evaluation, rinsing, and multilayer wound closure were performed. The postoperative treatment regimen depended on the approach used, the surgeon’s preference, and the extent of revision. Full weight bearing was allowed in n = 13 of cases, and partial weight bearing with 15 kg for 6 weeks was prescribed in n = 17 patients and with 15 kg for 8 weeks in n = 1 patient. For the posterior approaches, hip flexion was limited to 70° for 6 weeks. 

### 2.3. Clinical Evaluation

Patient demographics, comorbidities (Charlson comorbidity score) [24], and indications, type, and approach for both primary and revision THA were extracted from the institutional clinical information system (CIS) medical reports. In addition, patient-reported outcome measures (PROMs) were considered, including the modified Harris Hip Score (mHHS) [25,26], pain and satisfaction scores assessed using the numerical rating scale (NRS) [27], and the categorization of patient activity level using the University of California Los Angeles Activity Score (UCLA) [28,29], to assess the function before surgery and at the last follow-up. 

The mHHS is a reliable and valid tool that focuses on the patient’s pain, functional status, and activities of daily living before and after THA [26]. The mHHS was adapted as described by Byrd and Jones [25] by excluding the 9 deformity points and then multiplying the total score by a correction factor of 1.1 to give a maximum score of 100 (actually 100.1) [25]. The outcome can be interpreted as follows: <70 = poor; 70–80 = fair; 80–90 = good; and 90–100 = excellent [30]. The NRS is valid, reliable, and appropriate for use in assessing pain or satisfaction by providing sensitive data that can be statistically analyzed [27]. Both pain (0 = no pain; 10 = maximum pain) and satisfaction (1 = very satisfied; 5 = very dissatisfied) were assessed before and after surgery. The UCLA is a valid instrument for measuring the change in self-reported physical activity (1 = low; 10 = high) [28,29].

### 2.4. Radiographic Evaluation

Anteroposterior view and axial radiographs of the proximal femur at the last follow-up were compared with the immediate postoperative images. All radiographic parameters were assessed by two independent surgeons. 

The extent of femoral bone loss can be a substantial limitation in achieving rigid femoral fixation of the implant and the bone. Bone loss was graded according to the Paprosky classification [31]. 

Stem fixation was classified as stable or unstable: stable was defined as a stem with no progressive migration and minimal or no radiolucent lines around the stem, and unstable was defined as progressive subsidence or migration and divergent radiolucent lines surrounding the stem according to the Gruen zones [32]. Stem subsidence was measured as the distance from the tip of the greater trochanter to the stem shoulder [33]; stem subsidence > 10 mm, was considered as significant [34]. Distal cortical hypertrophy was calculated as the ratio of the width of the cortical and cancellous bone to the outside diameter of the femoral shaft at a level 1 cm distal to the inferior margin of lesser trochanter [35]. Heterotopic ossification was classified into four stages according to the criteria of Brooker et al. [36]. Additionally, fractures (intra- and postoperative), dislocations, and infections were analyzed. 

### 2.5. Outcomes

The primary endpoint was the clinical outcome evaluating the mHHS [30,37,38]. Secondary outcomes included pain and satisfaction (NRS) [27], patient activity (UCLA) [28,29], and radiographic outcomes as described above. 

### 2.6. Statistical Analyses

All analyses were performed with SPSS version 29 (IBM Corporation, Armonk, NY, USA). Postoperative PROMs at the last follow-up were compared with the preoperative values using the Wilcoxon test. Group differences were tested using the Mann–Whitney U test. Effect sizes were calculated to assess the relevance of a result and interpreted according to Cohen as small (d < 0.3), intermediate (d = 0.3–0.5), and large (d > 0.5) [39,40]. Continuous variables are expressed as the median and interquartile range, and categorical variables are expressed as frequencies. *p* values below 0.05 were considered to indicate significant differences.

## 3. Results

**Figure 1:**
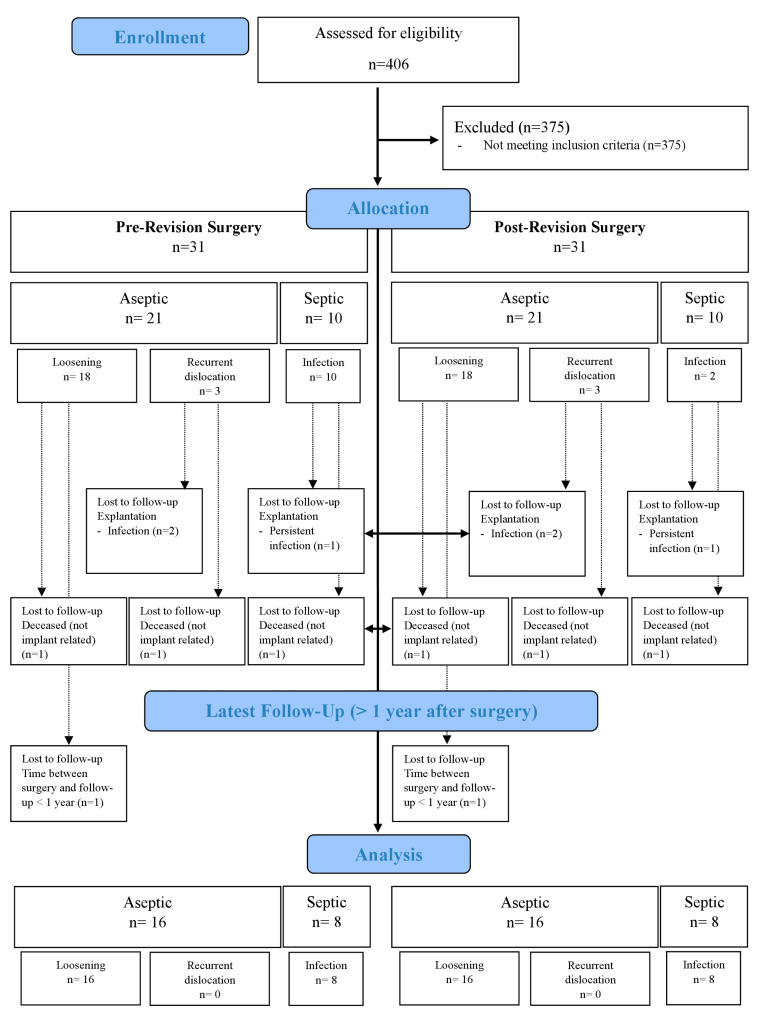
Study population throughout the study.

Of the 31 patients included, n = 24 could be analyzed at the last follow-up. Three patients were excluded with stem explantation due to persistent infection (n = 1) or suspected new infection (n = 2). Three additional patients died of nonimplant related reasons between revision surgery and follow-up. One patient had to be excluded because the time between surgery and follow-up was less than 1 year at the time of the study. The sample was divided into two subgroups according to the indication for the revision: aseptic (n = 16) and septic (n = 8). An overview of the study population is shown in Figure 1.

At the time of rTHA, the median (interquartile range) patient age and body mass index (BMI) of the 12 female (39%) and 19 male (61%) patients were 68.2 (61.2–78.4) years and 26.7 (24.6–29.4) kg/m^2^, respectively. Patient characteristics, comorbidities and indications, approach and type for THA and rTHA are shown in Table 1. The mean (standard deviation) lifetime between THA and rTHA was 6.1 (7.3) years, ranging up to 28.6 years. The mean prosthesis lifetime was significantly longer in patients with aseptic indication compared to patients with septic indication (septic: 8.3 (7.8) years; aseptic: 1.8 (7.2) years; *p* = 0.025).

### 3.1. Clinical Outcomes

All clinical outcome measures improved significantly at follow-up compared to the preoperative conditions (*p* < 0.001) with large effect sizes ranging from d = 2.852 to d = 1.761. The median (interquartile range) mHHS increased from a preoperative value of 24.2 (15.4–52.8) points to 80.9 (58.6–93.5) points at follow-up (*p* < 0.001). The NAS pain decreased from 7.5 (5.3–8.8) to 1.0 (0–2.8), and the NAS satisfaction decreased from 5.0 (3.0–5.0) to 2.0 (1.0–3.0) (*p* < 0.001). The activity level increased from UCLA 2.0 (2.0–5.5) to 6.0 (4.0–7.0). There were no significant differences between the aseptic and septic disease history, with mostly small effect sizes below d = 0.3, except for the postoperative mHHS (d = 0.383) and preoperative NAS pain (d = 0.431), which showed intermediate effect sizes. Descriptive statistics and comparisons between both pre- and post-rTHA and between the two subgroups (aseptic and septic) are shown in Table 2.

### 3.2. Radiographic Outcomes

The femoral bone loss types were Paprosky type I (n = 23) or type II/III (n = 8) defects. Stem fixation was stable in all of the patients. The mean stem subsidence was 0.5 mm (range 0–10 mm); n = 22 (92%) with no progressive subsidence, 3 mm in n = 1 (4%), and 10 mm in n = 1 (4%) caused by a trochanter major fracture. New cortical hypertrophy after revision was not observed in any of the hips. Other complications included heterotopic ossification Brooker stage III in n = 2 hips (8%), intraoperative femur perforation in n = 1 hip (4%), another periprosthetic fracture Vancouver type A in n = 1 hip (4%), and dislocations in n = 2 hips (8%). All radiographic results are shown in Table 3.

## 4. Discussion

Due to expanded indications, increased life expectancy, and the overwhelming success of primary THA, the numbers and need for rTHA are expected to increase [10,11,17]. Focusing on the time between THA and rTHA in our patients, which averaged 6.1 years and ranged to a maximum of 28.6 years, we had both long-lasting primary THAs with aseptic loosening and short- to medium-term revisions with implant-related or infection problems. Implantation of a short femoral stem in rTHA may preserve femoral bone for further revisions and reduce the stress shielding of the proximal femur, as has been demonstrated with the use of short stems in primary THA [4]. However, it should be a safe, long lasting, and quality-of-life improving treatment option. 

We observed a good clinical outcome in our rTHA patients with short stems: subjective hip function, pain, satisfaction and activity levels improved significantly from pre- to postoperative levels. This is consistent with the results of previous studies [41]. Liu et al. compared short stem with standard or long stem fixation in revision surgery and reported comparable mean hip function by HHS scores of 85.36 ± 12.43 for both revision approaches [42]. A comparison of the two subgroups “aseptic” and “septic” showed no difference in knee function, pain rating, or satisfaction: the indication for rTHA does not seem to have any influence on the PROMs after surgery. 

**Figure 2:**
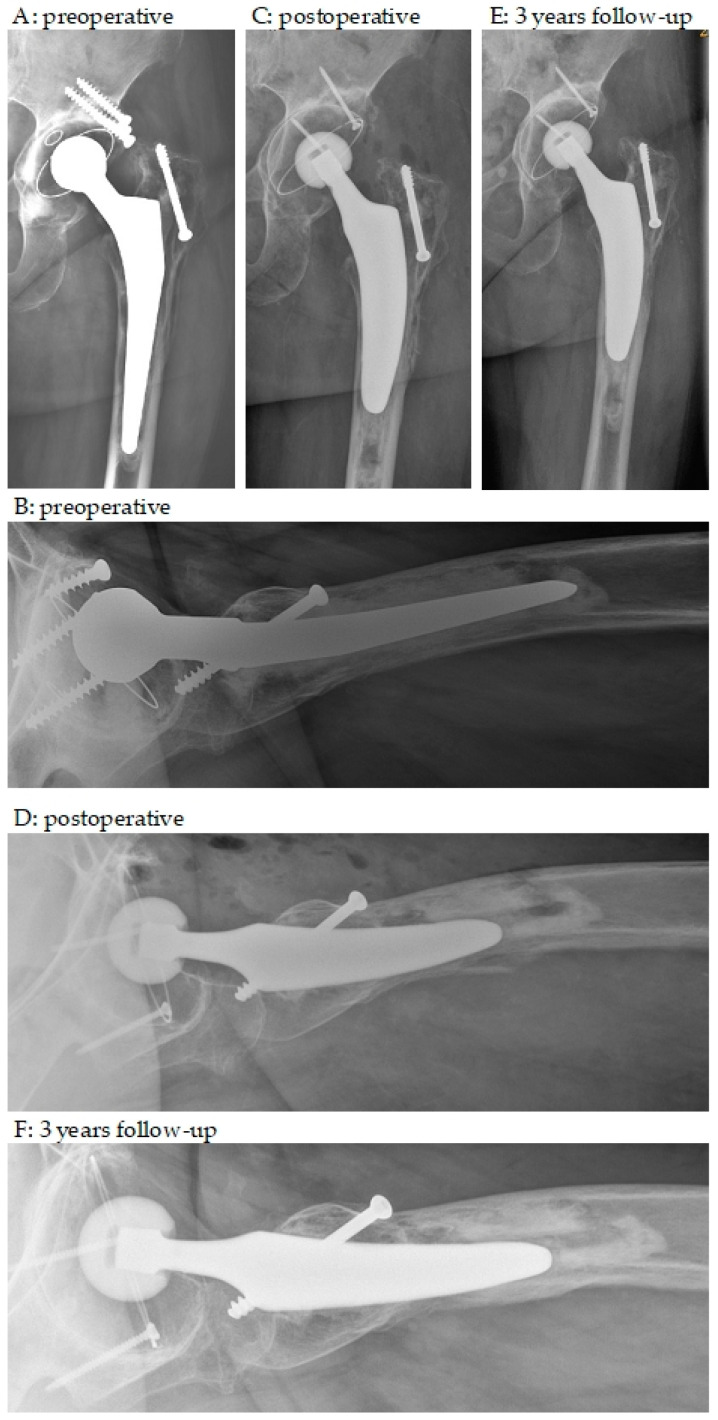
Anteroposterior and axial X-rays. (**A**,**B**) preoperative; (**C**,**D**) postoperative; (**E**,**F**) 3 year follow-up. A 79-year-old woman with aseptic loosening of a cemented stem and cup and eccentric PE wear; 27 years after primary THA; cup and stem revision with pressfit monobloc cup, additional screw fixation and cementless short stem; intraoperative posterior femoral perforation during cement removal with weak bone; fearing further perforation without osteotomy distal cement plug was left in place; last follow-up with healed bone and fixed short stem in place.

Our implant-related complication rate was 19% and included periprosthetic fractures, dislocations, infections, and ossifications. Both the complication rate and the type of complications confirmed previously published results in hip revision surgery [41,42]. The evidence to date suggests that short stems are a good alternative to standard or long stems in patients with good bone quality [4]. In contrast, previous studies have shown that age, osteoporosis, and intraoperative periprosthetic femoral fractures during rTHA are independent risk factors for early failure in patients undergoing revision surgery with short stems [42]. In contrast, only one intraoperative fracture (femoral perforation) occurred in our patient population and cannot be confirmed as a risk factor. The radiographs of this patient are shown in Figure 2.

It is important to note that all complications have different characteristics, and each requires individualized preoperative planning to determine the most appropriate treatment option. A closer analysis of the individual patient histories shows that patients who underwent revision for aseptic loosening had lower complication rates, and short stems seem to be applicable for these patients: of the 16 patients, one had an intraoperative fracture, one had a periprosthetic fracture (Vancouver type A), and two had dislocations. 

In contrast, the use of a short stem should be used with caution in patients with complex problems. As with other revisions, intraoperative complications occurred, requiring longer surgical times and causing early postoperative problems such as fractures or dislocations, which in turn were associated with swelling and immobilization and were often the beginning of wound healing and septic complications. Three patients had to be excluded from the final analysis due to new or persistent infections following intraoperative or early postoperative complications, and the stem was subsequently explanted. It is therefore imperative to ensure good surgical practice with correct cup position, stem rotation, and ligament balancing. In our data, patients with revisions due to infection had an increased rate of complications, which were recurrent infections, ossifications, or dislocations. 

**Figure 3:**
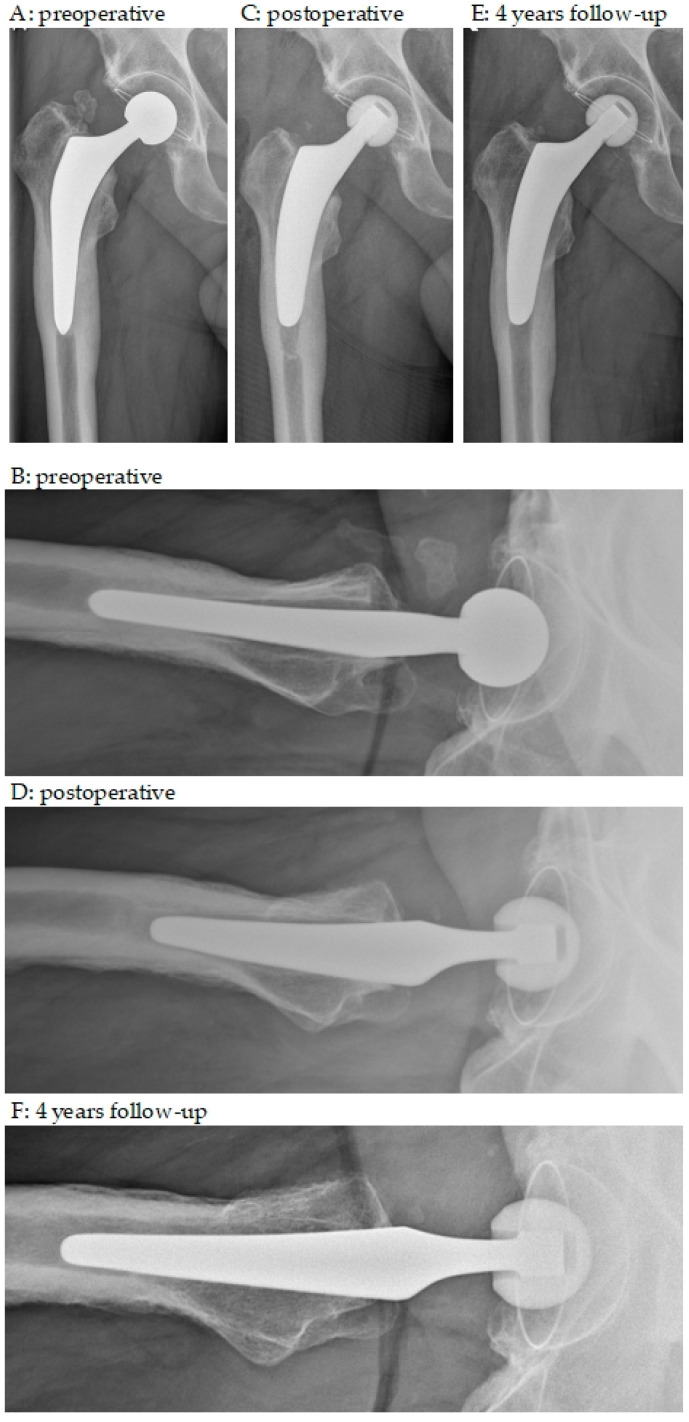
Anteroposterior and axial X-rays. (**A**,**B**) preoperative; (**C**,**D**) postoperative; (**E**,**F**) 4 years follow-up. A 66-year-old male with increasing pain and immobilization 5 years after THA; progressive stress shielding distally around the stem and loosening zone according to Gruen I, II and VI, VII as well as VIII, IX and XIII, XIV; no infection parameters and negative joint aspiration; stem revision with cementless short stem; in particular, in the axial view, the broader geometry of the short stem compared to the explanted stem allows sufficient bone stock and provides adequate rotational stability.

**Figure 4:**
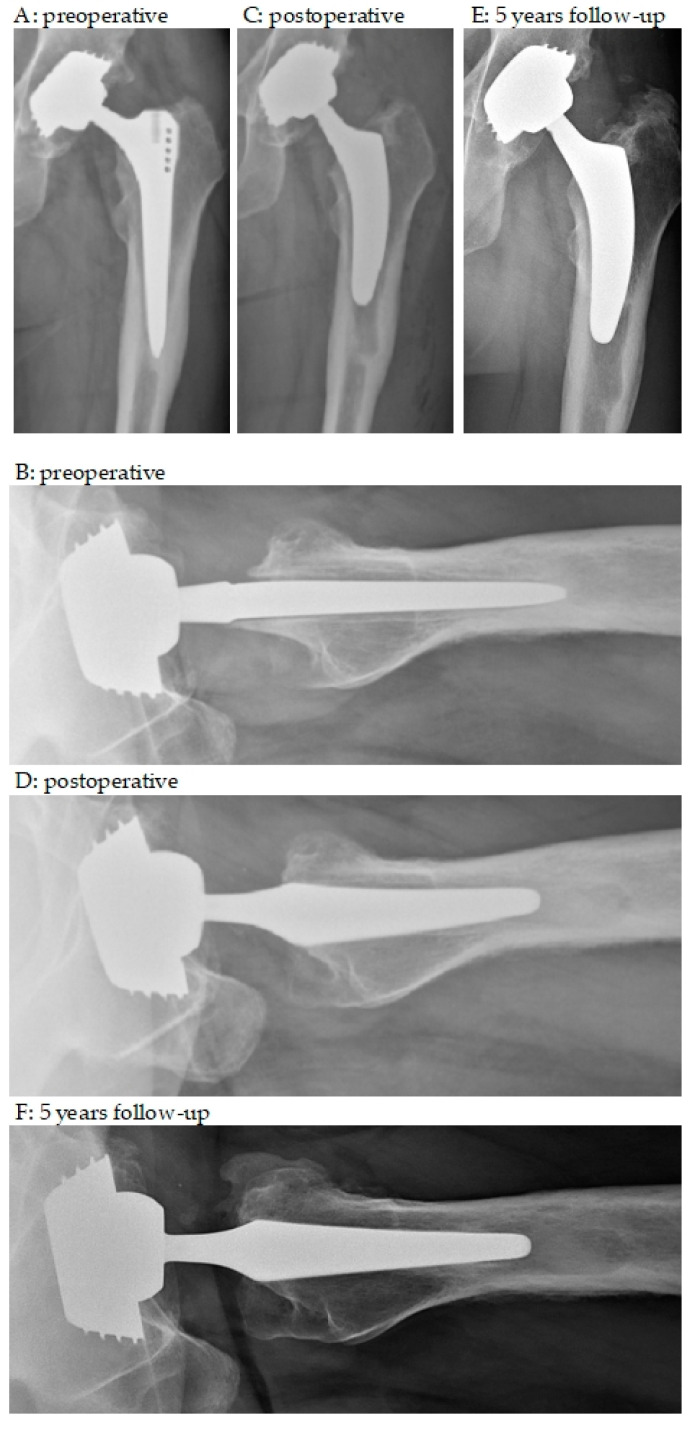
Anteroposterior and axial X-rays. (**A**,**B**) preoperative; (**C**,**D**) postoperative; (**E**,**F**) 5 years follow-up. A 52-year-old male with increasing pain and immobilization 6 years after THA; progressive stress shielding distally around the stem and loosening zone according to all Gruen zones; caused by aseptic loosening stem revision from a tapered rectangular stem to an short stem; in axial view, the relatively broad shoulder region compared to the explanted stem allows sufficient bone stock and provides superior rotational stability; from the first to last follow-up even new re-ossifications of the persisting loosening zones around the mid-stem.

Several models of short femoral stems are available, varying in terms of the insertion technique, osteotomy, and stem length [37]. The short stem used in the present study (Optimys^®^, Mathys, Switzerland) can be anchored metaphyseally or more diaphyseally depending on the individual alignment according to the patient’s anatomy [38]. A major advantage is the relatively broad shoulder region, which provides superior rotational stability compared to standard straight stems. This is illustrated by two cases in Figure 3 and Figure 4. Care should be taken to ensure good primary stability and not to undersize the implant. Consequently, limiting factors that may affect outcomes include elderly patients with comorbidities, known poor osteoporotic bone, extensive excised bone defects, and preoperative or intraoperative fractures involving the proximal femur.

### Strength and Limitations

This study provides information on the clinical and radiographic outcomes of rTHA using a short stem component. These results may assist surgeons in making decisions on implant selection. We employed a retrospective descriptive study design without a randomized controlled intervention. Only one type of short stem (Optimys^®^, Mathys, Switzerland) was implanted in patients with moderate bone defects (Paprosky type I and II/III); so, a limitation due to selection bias cannot be excluded. Finally, the sample consists of a rather heterogeneous study population with different indications and various comorbidities. This heterogeneity provides the opportunity to both selectively evaluate and distinguish between success and failure. These results should be validated by further experimental controlled studies.

## 5. Conclusions

We observed good clinical outcomes in our study population with the use of short femoral components in rTHA at up to 5 years of follow-up. Hip function as well as pain, satisfaction, and activity levels improved significantly from pre- to postoperative levels. 

Implant-related complications were comparable to those reported in the literature for rTHA, and the stability of the stem fixation demonstrated in our study supports the use of short stems in selective cases, i.e., patients with mild to moderate proximal bone deficiency, as in our study population. Caution should be exercised in patients with complex problems: septic patients had an increased rate of complications, such as recurrent infection, ossification, or dislocation.

Reducing the complexity of the revision with the advantages of short stems, especially the avoidance of diaphyseal bone stock, which is critical for further revisions, provides the experienced surgeon with another treatment tool in femoral stem revisions. Overall, our results support the consideration of short stems as a surgical option after a thorough preoperative analysis.

## Figures and Tables

**Table 1:** Patient characteristics.

Age at rTHA (years)	Median, interquartile Range 68.2 (61.2–78.4)
Sex	Number (%)
Female	12 (39%)
Male	19 (61%)
Body Mass Index (BMI, kg/m^2^)	Median, interquartile range 26.7 (24.6–29.4)
Comorbidities (Charlson Score/Index)	Median, interquartile range Score: 3 (2–5) Index: 10.3% (6.9–17.2%)
Indications for primary THA	Number (%)
Osteoarthritis	24 (77.4%)
Femoral neck fracture	3 (9.7%)
Femoral head necrosis	4 (12.9%)
Type of primary stem	Number (%)
Cemented	9 (29%)
Cementless	22 (71%)
Approach in primary THA	Number (%)
Anterior	16 (51.6%)
Anterolateral	1 (3.2%)
Lateral	13 (42.0%)
Posterior	1 (3.2%)
Indications for rTHA	Number (%)
Aseptic loosening	18 (58.0%)
Recurrent dislocation (aseptic)	3 (9.7%)
Infection	10 (32.3%)
Approach in rTHA	Number (%)
Anterior	6 (19.3%)
Lateral	15 (48.4%)
Posterior	10 (32.3%)
Components in rTHA	Number (%)
Stem only	8 (25.8%)
Stem and cup	23 (74.2%)
Time between primary THA and rTHA (years)	Median, interquartile range 2.7 (0.6–9.3)
Time between rTHA and last follow-up (years)	Median, interquartile range 2.3 (1.2–3.7)

**Table 2:** Clinical outcomes.

Parameter	Preoperative rTHA Median (Interquartile Range)	Postoperative rTHA Median (Interquartile Range)	Wilcoxon Test		Effect Size
			z-Value	*p*-Value	Cohen’s *d*
mHHS (All) (n = 24)	24.2 (15.4–52.8)	80.9 (58.6–93.5)	−4.011	<0.001	2.852
HHS (Aseptic) (n = 16)	34.1 (19.8–52.8)	89.7 (58.9–96.0)			
HHS (Septic) (n = 8)	22.0 (3.9–66.8)	69.3 (57.8–90.8)			
Mann–Whitney U Test					
z-value	−0.615	−0.922			
*p*-value	0.538	0.357			
Effect size *	0.253	0.383			
NAS (Pain) (All) # (n = 24)	7.5 (5.3–8.8)	1.0 (0.0–2.8)	−4.023	<0.001	2.878
NAS (Pain) (Aseptic) (n = 16)	8.0 (6.3–9.0)	1.0 (0.0–2.8)			
NAS (Pain) (Septic) (n = 8)	7.0 (2.8–8.0)	0.0 (0.0–4.3)			
Mann–Whitney U Test					
z-value	−1.031	−0.648			
*p*-value	0.302	0.517			
Effect size *	0.431	0.267			
NAS (Satisfaction) (All) † (n = 24)	5.0 (3.0–5.0)	2.0 (1.0–3.0)	−3.698	< 0.001	2.302
NAS (Satisfaction) (Aseptic) (n = 16)	5.0 (3.3–5.0)	2.0 (1.0–3.5)			
NAS (Satisfaction) (Septic) (n = 8)	5.0 (2.3–5.0)	2.0 (1.0–3.0)			
Mann–Whitney U Test					
z-value	−0.106	−0.227			
*p*-value	0.916	0.821			
Effect size *	0.043	0.093			
UCLA (All) (n = 24)	2.0 (2.0–5.5)	6.0 (4.0–7.0)	−3.237	0.001	1.761
UCLA (Aseptic) (n = 16)	2.0 (2.0–6.0)	6.0 (3.3–7.0)			
UCLA (Septic) (n = 8)	2.5 (2.0–5.5)	5.5 (4.0–6.8)			
Mann–Whitney U Test					
z-value	−0.291	−0.124			
*p*-value	0.711	0.901			
Effect size *	0.119	0.051			

* Effect size: Cohen’s *d*, # Pain (0 = no pain; 10 = maximum pain), † Satisfaction (1 = very satisfied; 5 = very dissatisfied).

**Table 3:** Radiographic results (n = 24).

Parameter	Number (%)
Stem fixation	
stable	24 (100%)
Stem subsidence	
<10 mm	1 (4%)
≥10 mm	1 (4%)
New cortical hypertrophy	0 (0%)
Heterotopic ossification	
Brooker ≤ I	17 (71%)
Brooker II	5 (21%)
Brooker III	2 (8%)
Fractures	
intraoperative (femur perforation)	1 (4%)
postoperative (Vancouver type A)	1 (4%)
Dislocation	2 (8%)
Infection	0 (0%)

## Data Availability

The data presented in this study are available on request from the corresponding author. The data are not publicly available due to ethical restrictions.
